
JOURNAL INFORMATION
==============================
NLM Title Abbreviation: Eur Psychiatry
Journal ID: Eur Psychiatry
NLM Journal ID: 9111820
Journal ID: EPA

European Psychiatry

ISSN: 0924-9338
EISSN: 1778-3585
Publisher: Cambridge University Press

ARTICLE INFORMATION
==============================
PMCID: PMC9412708
DOI: 10.1192/j.eurpsy.2020.7
Article ID: S0924933820000073
Article version: 1
Subjects: Abstracts

Ecp Programme

Publication date: 2020 Jul
Electronic publication date: 2020 Sep 4
Volume: 63
Issue: Suppl 1
Issue title: Abstracts of the 28th European Congress of Psychiatry
First page: S590
Last page: S593
Copyright: © The Author(s) 2020
Copyright year: 2020
Copyright holder: The Author(s)
License: This is an Open Access article, distributed under the terms of the Creative Commons Attribution licence (http://creativecommons.org/licenses/by/4.0/), which permits unrestricted re-use, distribution, and reproduction in any medium, provided the original work is properly cited.
License URL: https://creativecommons.org/licenses/by/4.0/

Page count: 4

==============================

## ECP Workshop: ABCS of psychotherapy

## Abstracts

### ECP001

### Abcs of psychotherapy

L. Claes[1,2]

I'm certainly willing to give a workshop on the abc's of Psychotherapy, I'm trained as a licensed CBT therapist. I would like to illustrate the abc's of psychotherapy by a case of a self-harming patient with comorbid personality problems and then show colleagues (a) how important it is to work on a good work alliance, (b) how important it is to perform good assessment, (c) to formulate a strong case formulation, (d) to link the chosen interventions to the elements of the case formulation and (c) to evaluate the effect of the interventions.

**Conflict of interest:** No

## ECP Symposium: mental health: better social environment for less inequality?

### ECP006

### Mental health disorders: an anthropological perspective

F. Thomas

At the heart of national and global mental health strategies lies an assumption that mental illness is a pathological condition that can be understood and dealt with through the use of demarcated diagnostic categories requiring distinct forms of biomedical or therapeutic intervention. Yet one only has to look to influential philosophical, religious and fictional texts across time to recognise that understandings of mental health and appropriate responses to the perceived afflictions of mental illness are socially determined within broader cultural and historical contexts. Drawing on research undertaken in the UK and Belarus, this presentation highlights how socially and culturally embedded norms, values and expectations influence the ways that mental illness is conceived and responded to. In the UK, focus is placed on understanding how diagnosis and treatment for common mental health disorders (depression, anxiety) have become bound up with broader political and economic agendas which fuel the pathologisation of poverty-related distress. In Belarus, focus is placed on the ways that economic and ideological changes have impacted on societal norms and expectations relating to mental health care, as well as on the lived experiences of families affected by mental illness. Such examples draw attention to the value of embedding an anthropological perspective in mental health research.

**Conflict of interest:** No

EUROPEAN PSYCHIATRIC ASSOCIATION

### ECP009

### Social adversity and neighborhoods: can we predict mental illness?

K. Mcdonald[1], T. Ding[2], D. Osborn[1], P. Wohland[3], P. French[4], P. Jones[5], G. Baio[6] and J. Kirkbride[7]*

**Background:** Providing timely, adequate and appropriately-resourced care to people experiencing first episode psychosis [FEP] needs to be informed by accurate prediction models of need.
**Methods:** We developed and validated a population-level prediction model of need for early intervention in psychosis [EIP] services using small-area level neighbourhood data in England up to 2025. Briefly, we combined small area incidence data on FEP, aged 16-64 years from empirical studies with prior information on psychosis risk by age, sex, ethnicity, deprivation and cannabis use. We tested six different Bayesian Poisson models for fit of this data to 2017 small-area populations of England, and compared our FEP predictions against observed national data. The best-fitting model was extrapolated to predict need for EIP care between 2019-2025, using small-area population projections.
**Results:** A model with age-sex interaction, ethnicity, small-area level deprivation, social fragmentation and regional cannabis use provided best internal and apparent validity, predicting 8112 (95% CrI: 7623-8597) FEP cases in England in 2017, compared with 8038 observed cases (difference: n=74; 0.94%). Apparent validity was acceptable at local levels, and by sex and ethnicity, although we observed greater-than-expected need before 35 years old. Predicted FEP cases rose between 2019-2025 by 6.2% to 9,541 new cases per year in line with demographic changes.
**Discussion:** Our translational epidemiological tool provides an accurate, validated method to inform mental health planners about future population need for psychosis care based on individual and small-area level determinants of need. Such tools can be used to enhance resource allocation in public mental health.

**Conflict of interest:** No

## Reached 2020 - but how online are we all, and how much more are we going to be?

### ECP014

### Virtual reality and the avatar

T. Craig

Virtual reality (VR) is increasingly being used in psychiatric research as a tool to assist psychological assessment and treatment. It enables researchers and clinicians to assess symptoms and functioning in an ecologically valid environment that being under the control of the clinician can be manipulated to titrate aspects of exposure that would be difficult if not impossible to achieve in the 'real' world. It has been used in the delivery of cognitive rehabilitation, social skills training interventions and psychosocial therapies for psychosis. AVATAR therapy is a non-immersive VR approach for helping patients manage distressing auditory verbal hallucinations. In involves a three-way interaction between therapist, patient and a computerised representation ('avatar') of their distressing hallucination. The patient sits in front of a monitor on which appears the avatar. The therapist, sitting in a separate room, can speak to the patient either as himself or as their hallucinated voice, adjusting what the avatar says according to the unfolding dialogue. The patient is encouraged to stand up to the avatar and through the dialogue, get to the point where it is no longer intimidating and may even become encouraging and supportive. Two pilot proof of concept studies and powered clinical trial comparing AVATAR therapy with a supportive counselling control intervention, have demonstrated effectiveness in terms of reduced frequency, omnipotence and associated distress of targeted AVH at 12 and 24 weeks. This presentation demonstrates the therapy, key research findings and outlines the design of a new clinical trial.

**Conflict of interest:** No

### ECP015

### Online therapy: does it work?

P. Cuijpers

It is well-established that treatments for depression and other common mental disorders can be effectively delivered online. In this presentation, the research on internet-based interventions for depression and anxiety will be summarized, including the results of a network meta-analysis in the which the different treatment formats of CBT for depression are examined. The effects of guided internet-based treatments are comparable to those of face-to-face therapies. In this presentation the presenter will also focus on questions like who can deliver internet-based treatments, who benefits more than others (based on individual patient data meta-analyses), whether unguided interventions are effective, and whether therapies should be based on CBT.

**Conflict of interest:** No

## ECP Workshop: Your attention please: let's talk about ADHD!

### ECP018

### Adhd in adults: diagnosis and treatment

J.A. Ramos-Quiroga

Attention deficit hyperactivity disorder (ADHD), which is characterized by the core symptoms inattention, impulsivity and hyperactivity, is a chronic neurodevelopmental disorder with childhood onset and persists into adulthood in many patients. A cross-national study conducted across Europe, the Americas, and Asia estimated a prevalence in adults of 3.4% according to DSM-IV-criteria. With release of DSM-5, specific diagnostic criteria for classification of adults are available. Comparing DSM-IV to DSM-5, for the clinician one of the most important changes is the requirement that some symptoms must be present before age 12 instead before age 7 and that the symptom threshold has been reduced from six to five symptoms. As diagnostic prevalence in psychiatric clinical samples and insurance records surveys is still far below the population based prevalence rate, next years will show, if these changes will help to improve patient care and recognition of adult ADHD. The focus of this lecture is nosology of ADHD including symptoms, functional impairment and to update diagnostic tools availables to diagnose ADHD in adults.

**Disclosure:** J.A.R.Q was on the speakers' bureau and/or acted as consultant for Eli-Lilly, Janssen-Cilag, Novartis, Shire, Lundbeck, Almirall, Braingaze, Sincrolab, Medice and Rubió in the last 5 years. He also received travel awards (air tickets + hotel) for taking p.

## ECP020

## How did we start our ADHD clinic and research from scratch?

I. Bitter

**Introduction:** Adult ADHD (aADHD) is an underrecognized and undertreated condition in Europe; no aADHD service and research was present in Hungary before 2005. This presentation will describe the establishment and some results of an internationally recognized aADHD clinic and research group (Group) in Budapest, Hungary.

**Method:** Publications of the Group will be reviewed – they responded to questions emerging about aADHD in the last 15 years. Some of the questions: what is the prevalence of aADHD in the world? Is it the same in Hungary? What is the rate of undiagnosed aADHD in psychiatric patients? What are the biological correlates of aADHD (focus on EEG)?

**Results:** Epidemiology – our metaanalysis (1) and local study (2) resulted in lower prevalence rates of aADHD than published in the early 2000s and similar to those widely accepted today. Comorbid aADHD is frequently not recognized (e.g. 3). High density EEG helped in describing a new region of interest and various deficits in aADHD (e.g. 4,5).

**Conclusions:** If motivation and a minimum of support available, high level clinical services and research can achieved within a short time for aADHD.

**References:** Simon V et al. British Journal of Psychiatry 194.3 (2009): 204-211. 2. Bitter I et al. European Archives of Psychiatry and Clinical Neuroscience 260.4 (2010): 287-296. 3. Bitter I et al. ADHD Attention Deficit and Hyperactivity Disorders 11.1 (2019): 83-89. 4. Czobor P et al. Brain Imaging and Behavior 11.6 (2017): 1616-1628. 5. Kakuszi B et al. Psychiatry Research: Neuroimaging 249 (2016): 57-66.

**Disclosure:** Dr. Bitter reports personal fees from Angelini , Eli Lilly,Janssen/Janssen-Cilag and Gedeon Richter outside of the submitted work; grants from the European Commission and European Group into Research in Schizophrenia.

## ECP021

## The silent minority: females with ADHD

O. Kilic[1]* and S. Young[2]

Research has identified gender differences in several correlates of ADHD. Firstly, population studies in childhood have shown a sex ratio of 1:3, suggesting that ADHD is less prevalent in girls than boys; however, in clinical studies, this ratio is between 1:5 to 1:9. In adulthood, the sex ratio of ADHD is 1:1 both in the population and clinical studies. Secondly, females with ADHD present more with inattention rather than hyperactivity and impulsivity. Symptoms of inattention are more subtle and likely to manifest in a structured environment such as high school or college, resulting in a delay in the diagnosis. Because of higher disruptiveness, boys are more likely to be referred to the clinic. Some researchers blame referral bias as the cause of gender differences in clinical settings and gender does not affect the clinical correlates of ADHD. Despite the available research, gender differences are poorly understood possibly because of the underrepresentation of females in the literature.

**Conflict of interest:** No

## Is there health without sexual health?

## ECP023

## The mental health professional as the guardian of sexual health

G. Pagkalos

A Mental Health provider, properly trained in Sexual Medicine, is essentially included in every multidisciplinary team specialized in the assessment and treatment of sexual health problems. Clients bring or would like to be asked on sexuality issues by Mental Health specialists. Sexual function, sexual interests and behaviors, sexual and gender identity issues are the most common sexuality themes of discussion with the Psychiatrist. In diagnostic manuals the sexual health issues were historically categorized in Mental Disorders. A new chapter on sexual health related conditions was added in the ICD-11, in order to de-pathologize sexuality concerns. Despite this, Psychiatrists are not exempt from their role to guide and care their patients' sexuality issues in order to facilitate the aim of medicine to promote wellness to every human. The bio-psycho-social model of treating sexual dysfunctions, the psychotherapeutic approaches of managing sexual interests and behaviors and the guidelines of gender transition procedures, involve the Mental Health Professionals in the holistic treatment of sexual health. The sexual dysfunctions being extremely prevalent in both general population and persons with mental illness should be routinely assessed by Psychiatrists. The psychological components and the distress caused often by sexual health problems require the involvement of a well-trained Specialist in treatment of mental disorders and relationship conflicts as maintaining factors of sexual health imbalance.These training opportunities for Mental Health Professionals are established worldwide in order to guarantee the highest level of education and, consequently, to ensure the proper skills to guard the sexuality as a fundamental part of life quality.

**Conflict of interest:** No

## ECP025

### What happens to your body and brain when you stop having sex?

M. Filip

One of the last things you're thinking about during sex is probably chemicals, your body and your brain—but they're more involved than you think. Many studies found that as many as 30 areas of the brain are active leading up to and after orgasm. Still, research on this topic is developing as it's a challenging topic to measure, test, and study. In my speech I would like to present everything researchers know so far about what happens to your brain and body during sex.

**Conflict of interest:** No

## Why Should Digital Psychiatry Be Taught to Psychiatric Trainees?

## W038

### A framework for teaching and considering informed decision making around digital mental health tools

J. Torous

Today clinicians and patients are using mobile technologies like apps and wearables. Numerous apps exists for serious mental illnesses, especially depression, and CBT ones are popular. Yet apps are not without risks such as breaches of privacy, offering incorrect information, frustrating patients with hard to use interfaces, and fracturing the therapeutic alliance as well as care. There is a need today for education to guide users away from such harmful apps and steer them to safe and effective tools. This session will teach the American Psychiatric Association's App Evaluation Framework using real world examples of apps for serious mental illness.

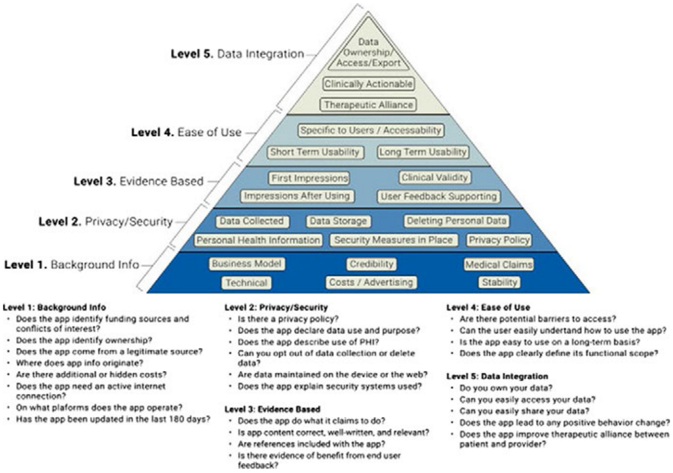

Participants will partake in hands on education to gain practical skills and competencies.

**Conflict of interest:** No

**Keywords:** mHealth; Technology; Digital

## W039

### Digital psychiatry – ready for an overview?

## Lecture Title:

M. Pinto Da Costa[1,2]

In recent years, there has been an explosion of interest in and research on the use of technology in mental health (ranging from telehealth, electronic health records and prescription, remote patient monitoring through smart-phone apps and sensors, virtual reality, robots and artificial intelligence). These technologies are intended to increase access, reduce disparities and costs, and have the potential to revolutionize mental health care. Early career psychiatrists should recognize and take advantage of the current state of new technologies to enhance mental health services. Therefore, the discussion amongst psychiatrists about the ever-changing landscape of technology in mental health care and research is needed. Different cases will be presented to spark reflection and discussion about the ethical implications of including technology in clinical practice and in research, currently and in the future. Matters as short- and long-term usefulness, sustainability, potential efficacy and safety, as well as privacy, financial and legal issues will also be debated. It is hoped that this talk will generate a lively discussion, gathering further understanding about digital psychiatry, its new boundaries and potential for scalability. The nature of the psychiatrist-patient relationship could be changed, as well as the future clinical practice in terms of diagnosis, follow-up, and treatment.

**Conflict of interest:** No


Article ID: ECP001
Subjects: ECP Workshop: ABCS of psychotherapy

Abcs of psychotherapy

Claes L. 1 2
1 University Antwerp , Faculty of Medicine & Health Sciences, Wilrijk , Belgium
2 University of Leuven , Psychology and Educational Sciences, Leuven , Belgium

Conflict of interest:

No

Article ID: ECP006
Subjects: Abstract, ECP Symposium: mental health: better social environment for less inequality?

Mental health disorders: an anthropological perspective

Thomas F.
University of Exeter , Wellcome Centre for Cultures and Environments of Health, Exeter , United Kingdom

Conflict of interest:

No

Article ID: ECP009

Social adversity and neighborhoods: can we predict mental illness?

Mcdonald K. 1
Ding T. 2
Osborn D. 1
Wohland P. 3
French P. 4
Jones P. 5
Baio G. 6
Kirkbride J. 7 *
1 University College London , Division Of Psychiatry, London , United Kingdom
2 University College London , Department of Statistical Sciences, London , United Kingdom
3 University of Queensland , School Of Earth And Environmental Sciences, Queensland , Australia
4 Greater Manchester West Mental Health Foundation, Psychosis Research Unit , Manchester , United Kingdom
5 University of Cambridge , Department of Psychiatry, Cambridge , United Kingdom
6 University College London , Department of Statistical Sciences, London , United Kingdom
7 University College London , Division Of Psychiatry, London , United Kingdom
* Corresponding Author:

Conflict of interest:

No

Article ID: ECP014
Subjects: Abstract; Reached 2020 - but How Online Are We All, and How Much More Are We Going to Be?

Virtual reality and the avatar

Craig T.
Kings College London, Institute of Psychiatry, Psychology and Neuroscience, Health Service and Population Research , London , United Kingdom

Conflict of interest:

No

Article ID: ECP015

Online therapy: does it work?

Cuijpers P.
Vrije Universiteit Amsterdam , Department of Clinical, Neuro and Developmental Psychology, Amsterdam , Netherlands

Conflict of interest:

No

Article ID: ECP018
Subjects: Abstract, ECP Workshop: Your attention please: let's talk about ADHD!

Adhd in adults: diagnosis and treatment

Ramos-Quiroga J.A.
Hospital Universitari Vall d'Hebron. Universitat Autònoma de Barcelona, Department of Psychiatry , Barcelona , Spain

Article ID: ECP020

How did we start our ADHD clinic and research from scratch?

Bitter I.
Semmelweis University , Department of Psychiatry and Psychotherapy, Budapest , Hungary

Article ID: ECP021

The silent minority: females with ADHD

Kilic O. 1 *
Young S. 2
1 Koç University Hospital , Department of Psychiatry, Istanbul , Turkey
2 Psychology Services Limited , London , United Kingdom
* Corresponding Author.

Conflict of interest:

No

Article ID: ECP023
Subjects: Abstract, Is There Health without Sexual Health?

The mental health professional as the guardian of sexual health

Pagkalos G.
Mental and Sexual Health Private Clinic, Sexual Health Department , Thessaloniki , Greece

Conflict of interest:

No

Article ID: ECP025

What happens to your body and brain when you stop having sex?

Filip M.
Polish Psychiatric Association, Specialty Training Section, Lodz , Poland

Conflict of interest:

No

Article ID: W038
Subjects: Abstract, Why Should Digital Psychiatry Be Taught to Psychiatric Trainees?

A framework for teaching and considering informed decision making around digital mental health tools

Torous J.
Beth Israel Deaconess Medical Center, Division of Digital Psychiatry , Boston , United States of America
Keywords: mHealth, Technology, Digital

Conflict of interest:

No

Article ID: W039

Digital psychiatry – ready for an overview?

Lecture Title:

Da Costa M. Pinto 1 2
1 Queen Mary University of London , Unit of Social and Community Psychiatry, London , United Kingdom
2 University of Porto , Institute of Biomedical Sciences Abel Salazar (ICBAS), Porto, Portugal
Keywords: Digital mental health, Early Career Psychiatrists, education, Technology

Conflict of interest:

No

